# Supplementary material for: Targeted mutagenesis of the herpesvirus fusogen central helix captures transition states
Source: Nat Commun. 2023 Dec 2;14:7958. doi: 10.1038/s41467-023-43011-w (PMC10693595; doi:10.1038/s41467-023-43011-w)
Supplement: Supplementary file 1 — Supplementary Information [file 41467_2023_43011_MOESM1_ESM.pdf]

Supplementary Information

For

Caught in the Act: targeted mutagenesis of the herpesvirus fusogen central helix captures transition states

Zhou et al.

Genus	Virus	Start	Sequence	End	Host species	NCBI accession
Varicellovirus	VZV	511-	SVEFAMLQFTYDHIQEHVNEMLARISSSWCQLQNRERALWSGLFPI	-556	Human	AGC94521
Varicellovirus	CeHV-9	501-	SVEFAMLQFTYDHIQEHVNEMLARISTSWCQLQNKERALWSSLFPV	-546	African green monkey	AAA58703
Varicellovirus	PRV	528-	SAEFARLQFTYDHIQAHVNDMLSRIAAAWCELQNKDRTLWGEMSRL	-573	Pig	ALB06102
Varicellovirus	EHV-1	574-	SIEFAMLQFAYDHIQSHVNEMLSRIATAWCTLQNKERTLWNEMVKI	-619	Horse	BAF43295
Varicellovirus	EHV-3	590-	SVEFAMLQFAYDHIQAHVNEMLSRIATAWCTLQNKERVLWNEMVKI	-635	Horse	YP_009054936
Varicellovirus	EHV-4	569-	SIEFAMLQFAYDHIQSHVNEMLSRIATAWCTLQNKERTLWNEMVKV	-614	Horse	QYL35303
Varicellovirus	EHV-8	573-	SIEFAMLQFAYDHIQSHVNEMLSRIATAWCTLQNKERTLWNEMVKI	-618	Horse	AUS94926
Varicellovirus	EHV-9	475-	SIEFAMLQFAYDHIQSHVNEMLSRIATAWCTLQNKERTLWNEMVKI	-520	Horse	YP_002333514
Varicellovirus	FeHV-1	538-	SVEFAMLQFAYDYIQAHVNEMLSRIATAWCTLQNREHVLWTETLKL	-583	Cat	ALJ85888
Varicellovirus	CaHV-1	472-	SVHFAMLQFAYDHIQSHVNEMLSRIATAWCNLQNKERTLWNEVMKL	-517	Dog	AEK27122
Varicellovirus	BoHV-1	535-	SAEFAALQFTYDHIQDHVNTMFSRLATSWCLLQNKERALWAEAAKL	-580	Cattle	QBI59636
Varicellovirus	BoHV-5	541-	SAEFAALQFTYDHIQDHVNTMFSRLATSWCLLQNKERALWAEAAKL	-586	Cattle	ARM56009
Varicellovirus	BuHV-1	545-	SAEFAALQFTYDHIQDHVNTMFSRLATSWCLLQNKERALWAEAAKL	-590	Buffalo	APO15888
Varicellovirus	CpHV-1	519-	SAEFAALQFTYDHIQDHVNAMFSRLATSWCLLQNKERALWAEAAKL	-564	Goat	YP_009664694
Varicellovirus	CvHV-1	530-	SAEFAALQFTYDHIQDHVNTMFSRLATSWCLLQNKERALWAEAAKL	-575	Deer	YP_009664695
Varicellovirus	CvHV-2	532-	SAEFAALQFTYDHIQDHVNAMFSRLATSWCQLQNKERTLWAEAAKL	-577	Deer	AVT50752
Varicellovirus	CvHV-3	534-	SAEFAALQFTYDHIQDHVNTMFSRLATSWCLLQNKERALWAEAAKL	-579	Deer	YP_010087575
Varicellovirus	MoHV-1	502-	GAEFAALQFTYNHIQSHVNEMFARIATAWCALQNKELALWKETVKL	-547	Beluga whale	YP_010084963
Varicellovirus	PhHV-1	478-	SIHFAMLQFAYDHIQSHVNEMLSRIATAWCNLQNKERTLWNEVMKL	-523	Seal	CAA92272
Simplexvirus	HSV-1	500-	SIEFARLQFTYNHIQRHVNDMLGRVAIAWCELQNHELTLWNEARKL	-545	Human	AFH41177
Simplexvirus	HSV-2	497-	SIEFARLQFTYNHIQRHVNDMLGRIAVAWCELQNHELTLWNEARKL	-542	Human	CAB06752
Simplexvirus	CeHV-2	485-	SVEFARLQFTYDHIQRHVNDMLGRIATAWCELQNRELTLWNEARRL	-530	African green monkey	CAA40256
Simplexvirus	AtHV-1	523-	TVEFARLQFTYDHIQKHVNEMLGRIAAAWCQLQNQELVLWNEARKL	-568	Spider monkey	YP_009361910
Simplexvirus	McHV-1	487-	SVEFARLQFTYDHIQRHVNDMLGRIAIAWCELQNHELTLWNEARKL	-532	Macaque monkey	NP_851887
Simplexvirus	PnHV-3	499-	SIEFARLQFTYNHIQRHVNDMLGRIAVAWCELQNHELTLWNEARKL	-544	Chimpanzee	YP_009011014
Simplexvirus	SaHV-1	509-	SVEFARLQFTYDHIQKHVNEMLGRIAAAWCQLQNQELVLWNEARKL	-554	Squirrel monkey	AAM22795
Simplexvirus	PaHV-2	488-	SIEFARLQFTYDHIQRHVNDMLGRIAIAWCELQNRELTLWNEARRL	-533	Baboon	BAE79592
Simplexvirus	BoHV-2	518-	SVEFARLQFTYNHIQKHVNEMFGRMAVSWCELQNQELTLWNEAKKI	-563	Cattle	YP_009664618
Simplexvirus	LeHV-4	482-	SVEFARLQFTYDHIQRHVNEMLGRLAVSWCELQNQELTLWNEAQKL	-527	Rabbit	YP_009230158
Simplexvirus	MaHV-1	483-	SIEFARLQFTYDHIQQHVNEMLGRIAIAWCELQNRELTLWNEARKL	-528	Parma wallaby	YP_009227260
Simplexvirus	MaHV-2	486-	SIEFARLQFTYDHIQQHVNEMLGRIAIAWCELQNRELTLWNEARKL	-531	Quokka	YP_009664622
Simplexvirus	PtHV-1	340-	SIEFARLQFTYDHIQKHVNDMLGRIAIAWCQLQNQELVLWNEARKL	-385	Fruit bat	YP_009042089
Mardivirus	AnHV-1	586-	SAQFAMLQYTYDHIQAHVNDMLSRIAVSWCELQNKESVLWAEMRKV	-631	Duck	AET71141
Mardivirus	CoHV-1	579-	SAQYAMLQFTYDHIQGHVNDMFSRIAVAWCELQNKERVLWSEALKI	-624	Pigeon	YP_009352935
Mardivirus	MeHV-1	463-	SVQFAMLQFLYDHIQAHINEMFSRIATAWCELQNKELVLWREAIKI	-508	Turkey	NP_073321
Mardivirus	GaHV-2	457-	SVQFAMLQFLYDHIQTHINDMFSRIATAWCELQNRELVLWHEGIKI	-502	Chicken	AAM97702
Mardivirus	GaHV-3	458-	SVQFAMLQFLYDHIQTHINDMFSRIATAWCELQNKELALWQEGMKI	-503	Chicken	NP_066859
Mardivirus	SpAHV-1	487-	TVHFAMLQFTYDHIQRHVNEMLGRIAKAWCELQNRESVLWREMRKM	-532	Penguin	YP_009342376
Iltovirus	GaHV-1	452-	SATFAMLQFAYDKIQAHVNELIGNLLEAWCELQNRQLIVWHEMKKL	-497	Chicken	CAA39573
Iltovirus	PsHV-1	470-	SATFAMLQFTYDKIQKHVNMLIGNLLEAWCEMQNRQLVVWHEIKKL	-515	Parrot	AAQ73706
Scutavirus	ChHV-5	431-	DATFTQVQFTYDVLRQHINSVFSRLVNAWCELQNRDVTVWELLLNI	-476	Sea turtle	QHH25918
Scutavirus	TeHV-3	427-	TAAFLQIQYTYDKLQAHNNAMFSRIVYAWCELQNRDITMWEQLNKI	-472	Tortoise	YP_009176910
			`   :  :*: *: :: * * ::..:  :** :**::  :*     :`			

Supplementary Fig. 1. Sequence alignment of the amino acids equivalent to VZV gB DIII central helix from the five genera of the subfamily alphaherpesvirinae. Virus host species and the glycoprotein gB NCBI accession are listed and the residues corresponding to ^{526}EHV^{528} are colored in blue. Conservation among residues is presented based on Clustal Omega: "." indicates weak similarity; ":" indicates strong similarity; "*" indicates full conservation. Histidine corresponding to H527 in VZV is highlighted in pink box. VZV: Varicella-zoster virus; CeHV-9: Cercopithecine alphaherpesvirus 9; PRV: pseudorabies virus; EHV-1: Equine alphaherpesvirus 1; EHV-3: Equine alphaherpesvirus 3; EHV-4: Equine alphaherpesvirus 4;

EHV-8: Equine alphaherpesvirus 8; EHV-9: Equine alphaherpesvirus 9; FeHV-1: Felid alphaherpesvirus 1; CaHV-1: Canid alphaherpesvirus 1; BoHV-1: Bovine alphaherpesvirus 1; BoHV-5: Bovine alphaherpesvirus 5; BuHV-1: Bubaline alphaherpesvirus 1; CpHV-1: Caprine alphaherpesvirus 1; CvHV-1: Cervid alphaherpesvirus 1; CvHV-2: Cervid alphaherpesvirus 2; CvHV-3: Cervid alphaherpesvirus 3; MoHV-1: Monodontid alphaherpesvirus 1; PhHV-1: Phocid alphaherpesvirus 1; HSV-1: Herpes simplex virus 1; HSV-2: Herpes simplex virus 2; CeHV-2: Cercopithecine alphaherpesvirus 2; AtHV-1: Ateline alphaherpesvirus 1; McHV-1: Macacine alphaherpesvirus 1; PnHV-3: Panine alphaherpesvirus 3; SaHV-1: Saimiriine alphaherpesvirus 1; PaHV-2: Papiine alphaherpesvirus 2; BoHV-2: Bovine alphaherpesvirus 2; LeHV-4: Leporid alphaherpesvirus 4; MaHV-1: Macropodid alphaherpesvirus 1; MaHV-2: Macropodid alphaherpesvirus 2; PtHV-1: Pteropodid alphaherpesvirus 1; AnHV-1: Anatid alphaherpesvirus 1; CoHV-1: Columbid alphaherpesvirus 1; MeHV-1: Meleagrid alphaherpesvirus 1; GaHV-2: Gallid alphaherpesvirus 2; GaHV-3: Gallid alphaherpesvirus 3; SpAHV-1: Spheniscid alphaherpevirus 1; GaHV-1: Gallid alphaherpesvirus 1; PsHV-1: Psittacid alphaherpesvirus 1; ChHV-5: Chelonid alphaherpesvirus 5; TeHV-3: Testudinid alphaherpesvirus 3.

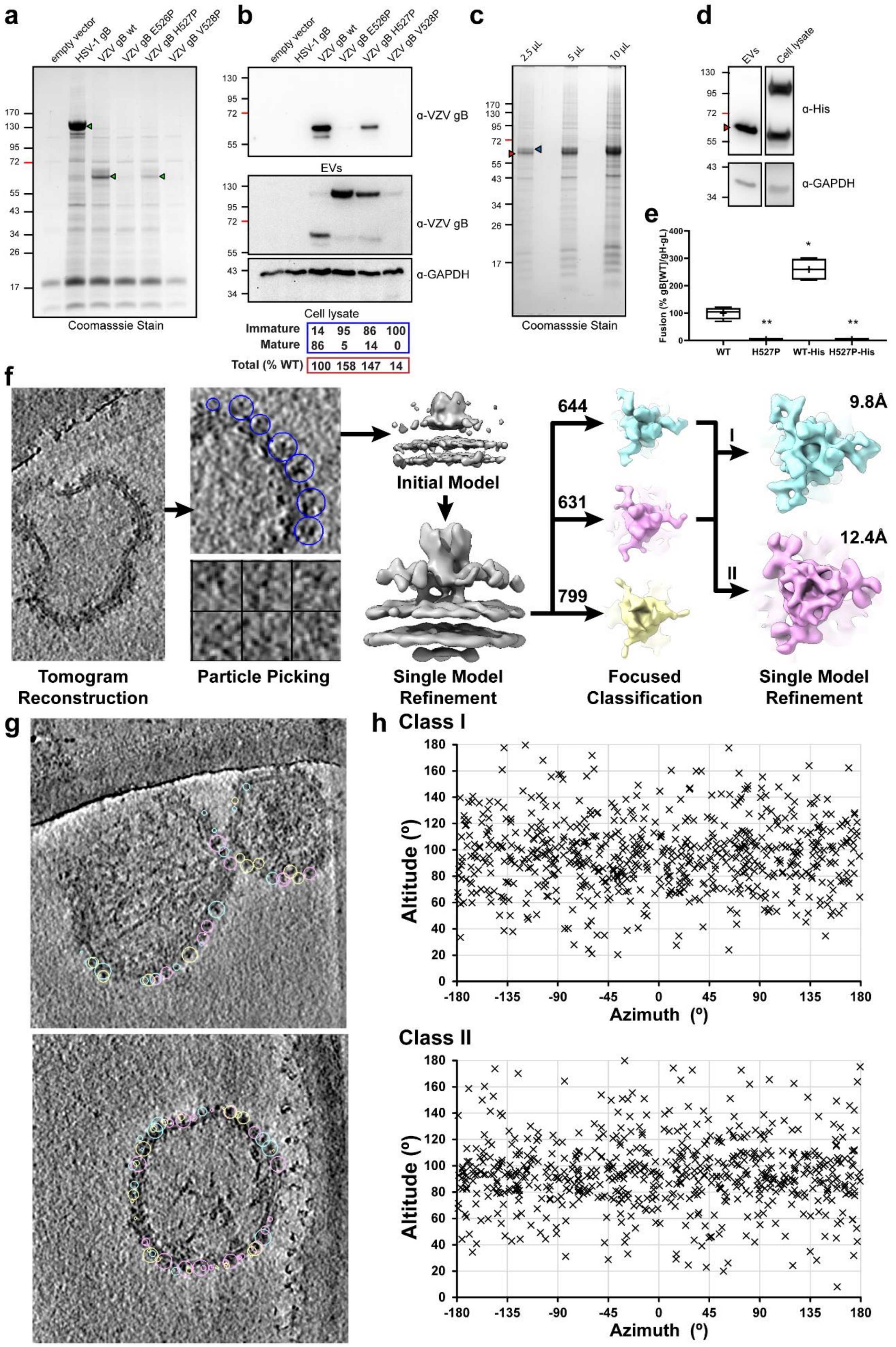
a
empty vector
HSV-1 gB
VZV gB wt
VZV gB E526P
VZV gB H527P
VZV gB V528P
Coomasssie Stain
b
α-VZV gB
EVs
α-GAPDH
Cell lysate
Immature 14 95 86 100
Mature 86 5 14 0
Total (% WT) 100 158 147 14
c
2.5 μL
5 μL
10 μL
Coomasssie Stain
d
EVs
Cell lysate
α-His
e
Fusion (% gB[WT]/gH-gL)
WT
H527P
WT-His
H527P-His
f
644
631
799
9.8Å
12.4Å
I
II
Initial Model
Tomogram Reconstruction
Particle Picking
Single Model Refinement
Focused Classification
Single Model Refinement
g
h
Class I
Altitude (º)
Azimuth (º)
Class II
Altitude (º)
Azimuth (º)

Supplementary Fig. 2. Characterization of EVs expressing gB proline substitutions to H527 residue in DIII. a Coomassie blue stain of EVs purified from BHK-21 cells transfected with vectors expressing VZV WT gB, gB ^{526}EHV^{528} proline mutants, HSV-1 gB or empty vector. Green arrow heads highlight the uncleaved form of HSV-1 gB and the cleaved form of VZV gB. **b** Western blot of EVs from (**a**) detected with rabbit anti-VZV gB 746-867 and whole cell lysates from transiently transfected BHK-21 cells; GAPDH loading control. The relative molecular abundance of the immature full-length, and the mature cleaved fragment of gB in the transfected cells were shown as a percentage of the sum abundance of the two gB forms (blue box); the combined band density of the immature full-length and the cleaved fragment of gB in cell lysates was normalized to that of GAPDH and shown as a percentage of WT gB (red box). **c** Coomassie blue stain of EVs purified from BHK-21 cells transfected with vectors expressing the C-terminally His-tagged gB[H527P] (H527P-His). The SDS-PAGE gel was loaded with increasing quantities of EVs. N-terminal and C-terminal cleavage products of VZV gB[H527P] are indicated by blue and red arrowheads respectively. **d** Western blot of EVs from (**c**) and cell lysates detected for gB (anti-His tag); GAPDH loading control; C-terminal cleavage product of VZV gB[H527P] is indicated by red arrowhead. Mass markers (kDa) are shown on the left (**a-d**). **e** Box and whisker plots for cell-cell fusion measured by the stable reporter fusion assay (SRFA) using CHO-DSP1 cells transfected with vectors expressing untagged (WT) or His-tagged WT gB (WT-His) or the corresponding H527P mutants (H527P and H527P-His) with gH-gL, and mixed with MeWo-DSP2 cells for 48hrs. Fusion efficiency was normalized to that mediated by gB[WT]/gH-gL (%). Box represents 25-75 percentile, whiskers extend to 10-90 percentile, the median is the horizontal band and the mean is the +. Data from n = 2 independent experiments is shown. The statistical difference compared to WT gB was analyzed by one-way ANOVA, Dunnett's multiple comparisons test (*, $p = 0.0159$; **, $p = 0.0067$-0.0069). **f** A representative of 43 tomogram reconstructions, particle picking, and classification scheme of gB[H527P] particles in EVs membranes identified in cryo-ET micrographs. Highlighted blue circles, initially yielded three classes (Cyan: Class I; Purple: Class II; Yellow: Class III). Further analysis dismissed Class III due to low resolution in the central region, finally yielding the two classes producing the Class I (9.8Å) and Class II (12.4Å) cryo-EM maps. Particle numbers for each class are shown for the Focused Classification. **g** Distribution of Class I to III particles on the surface of EVs. The circles represent manual particle picking for each class, color corresponding to each class shown in (**f**). **h** Angular distribution of the particles used to generate the Class I and Class II Cryo-EM maps. Source data are provided as a Source Data file.

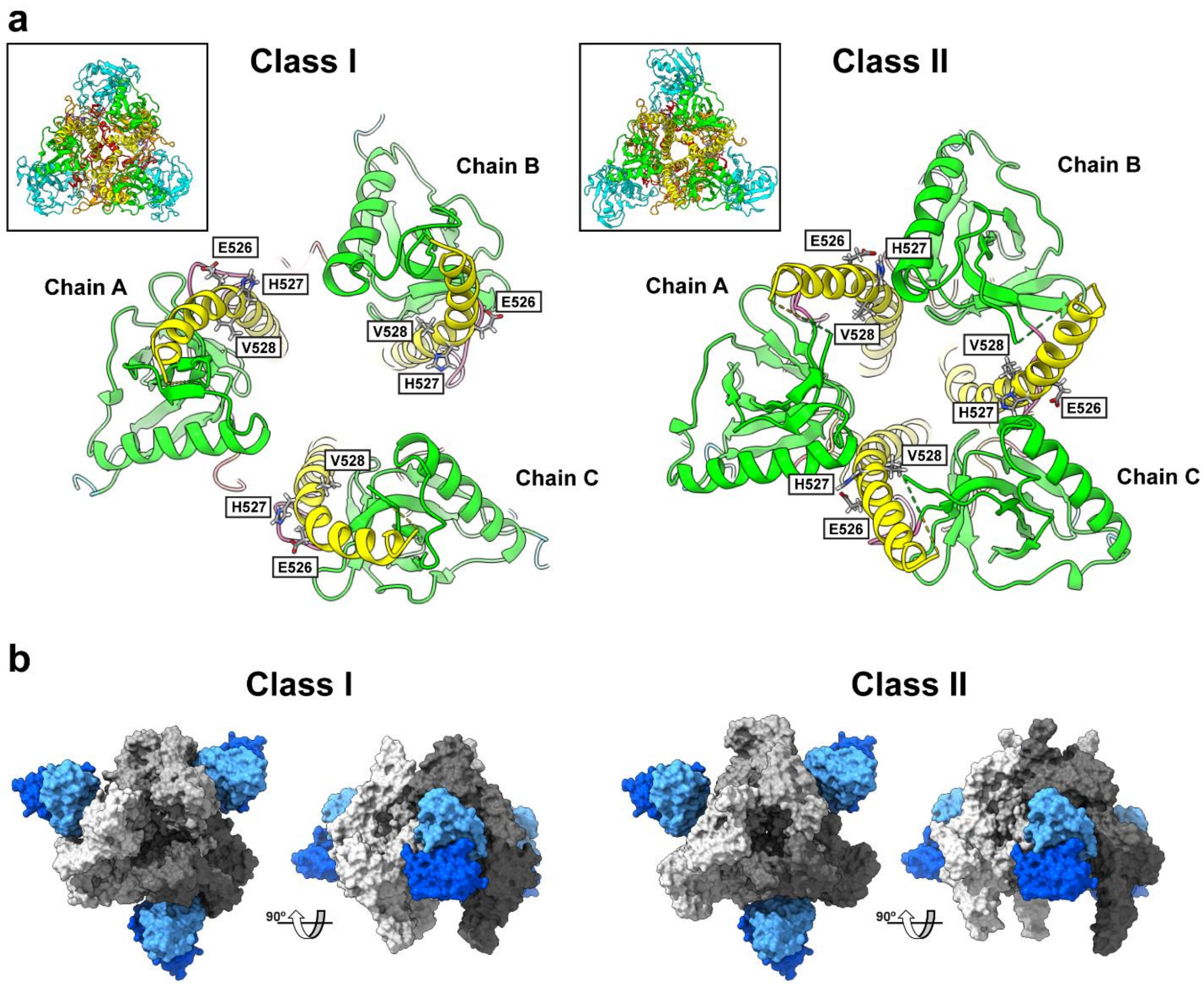

Supplementary Fig. 3. The location of residues ^{526}EHV^{528} and predicted binding of mAb 93k for Class I and Class II models of gB[H527P]. a Ribbon diagrams highlight the locations of the ^{526}EHV^{528} residues in the gB DIII central helix. The insets show the top views of the Class I and Class II models with each domain colored; DI (cyan), DII (green), DIII (yellow), DIV (orange), DV (red) and linker regions (hot pink). The main panels show an area of the Class I and Class II models focused on DII and the upper portion of the DIII central helix for each of the chains A to C. **b** Molecular surface representation demonstrating the location of human, VZV neutralizing mAb 93k fab fragments predicted to bind to the Class I and Class II models. The three chains of VZV gB are colored white (A), grey (B), and dark grey (C), and the heavy and light chains of the mAb 93k fab fragments are colored blue and light blue respectively. Fab fragments of mAb 93k bind to VZV gB DIV [1,2].

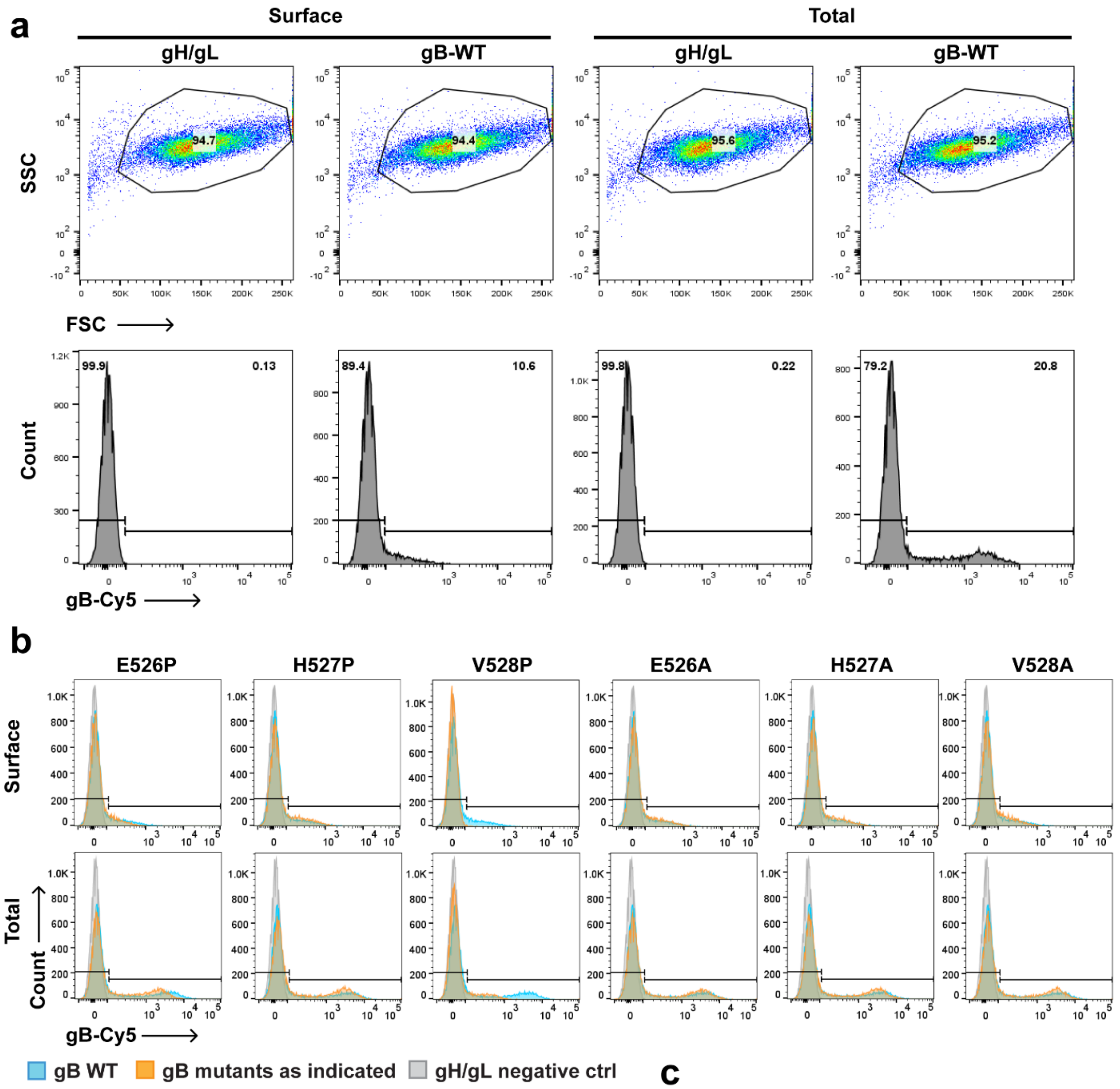

	Frequency of postive cells (%WT)		Median fluorescence intensity (%WT)	
	Surface	Total	Surface	Total
E526P	80.5 ± 3.3	109.0 ± 14.9	ND	70.3 ± 8.9
H527P	119.3 ± 16.6	118.1 ± 14.7	ND	95.4 ± 3.6
V528P	3.9 ± 0.7	48.0 ± 8.2	ND	17.5 ± 0.6
E526A	119.6 ± 3.6	118.8 ± 3.7	ND	109.6 ± 0.7
H527A	110.8 ± 5.2	120.9 ± 6.3	ND	98.1 ± 3.6
V528A	104.4 ± 6.0	120.6 ± 2.4	ND	98.5 ± 6.8

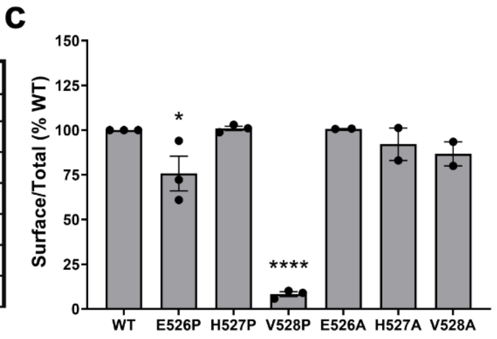

Supplementary Fig. 4. The analysis of gB expression by flow cytometry. a Gating strategy for the quantification of gB levels using mAb 93k in flow cytometry. The representative samples are for total and surface staining of CHO-DSP1 cells transfected with gH/gL or gB and detected with the anti-gB mAb Cy5-conjugated 93k. The top panels represent examples of CHO cells nonpermeabilized for surface, or permeabilized for total, gated using forward scatter (FSC) and side scatter (SSC). The lower panels represent the histograms from the gated CHO cells and show the specificity of the 93k mAb for VZV gB. **b** Surface or total expression of WT gB or gB mutants. The representative samples show the population of WT gB-expressing cells (blue) and mutant gB-expressing cells (orange) was gated against cells transfected with gH-gL (grey). The frequency of gB-expressing cells and median of fluorescence intensity (MFI) were compared to that in the WT gB and presented as percentage of WT in the table (%WT; mean ± SD). **c** The ratio of surface to total gB expression from **b** normalized to WT gB (% WT) (mean ± SEM) was calculated. n = 3 independent experiments for WT gB and proline mutants, and n = 2 independent experiments for alanine mutants. Statistical differences were evaluated by one-way ANOVA, Dunnett's multiple comparisons test (*, $p = 0.0272$; ****, $p < 0.0001$). Source data are provided as a Source Data file.

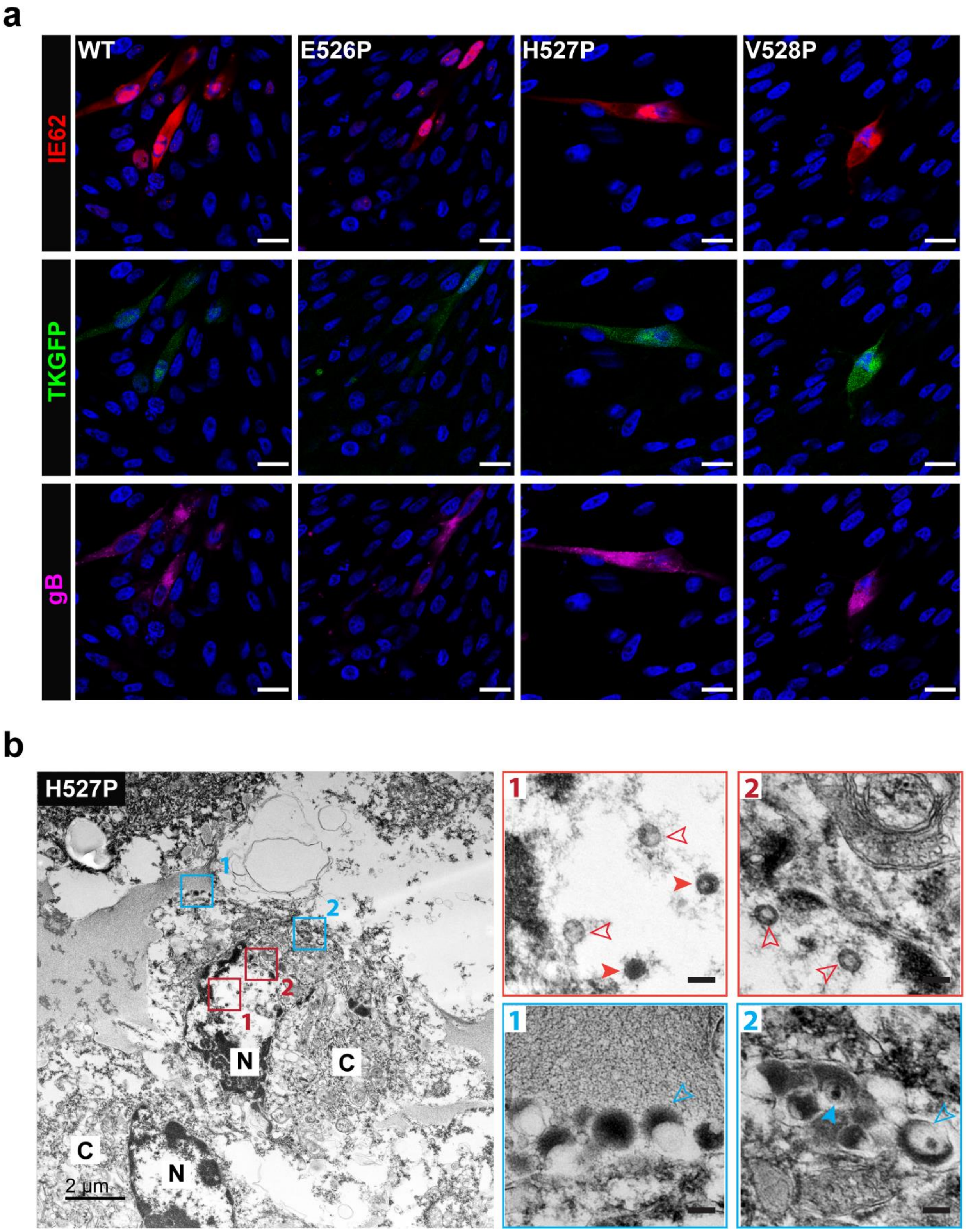
a
WT
E526P
H527P
V528P
IE62
TKGFP
gB
b
H527P
1
2
N
C
C
N
2 μm

Supplementary Fig. 5. Proline substitutions at residues ^{526}EHV^{528} in DIII does not interfere with gB expression and virus assembly in BAC transfected cells. a Confocal microscopy images of MeWo cells transfected with pOka-TKGFP (WT), pOka-TKGFP-gB[E526P] (E526P), pOka-TKGFP-gB[H527P] (H527P), or pOka-TKGFP-gB[V528P] (V528P) BACs at 72 hrs post transfection. All the BACs expressed thymidine kinase (TK) fused with GFP fluorescence protein at the C-terminus. Cells were stained for immediate-early protein (IE62; red), gB (93k; violet), and nuclei (Hoechst 33342; blue). Representative examples from 5 fields of view recorded from n = 2 independent experiments are shown. Scale bar = 20 µm. **b** Transmission Electron Microscopy (TEM) images of MeWo cells transfected with pOka-TKGFP-gB[H527P] BAC at 48 hrs post transfection. The transfected cells were harvested at 48 hrs post transfection and enriched for GFP-expressing cells by sorting prior to TEM. Panels at the right are magnifications of areas indicated by boxes in the larger images (N: nuclei; C: cytoplasm). Boxes outlined in red show representative viral capsids, with solid red arrowheads indicating viral DNA containing C-capsid and open red arrowheads indicating empty A-, or scaffold containing B capsids. Boxes outlined in blue show representative viral particles, with solid blue arrowhead indicating complete virus particle in the process of assembly in the cytoplasm and open blue arrowheads indicating light particles on the cell surface or in the process of assembly in the cytoplasm. Assay efficiency is provided in Supplementary Table 4. In the magnified images, scale bar = 0.1 µm.

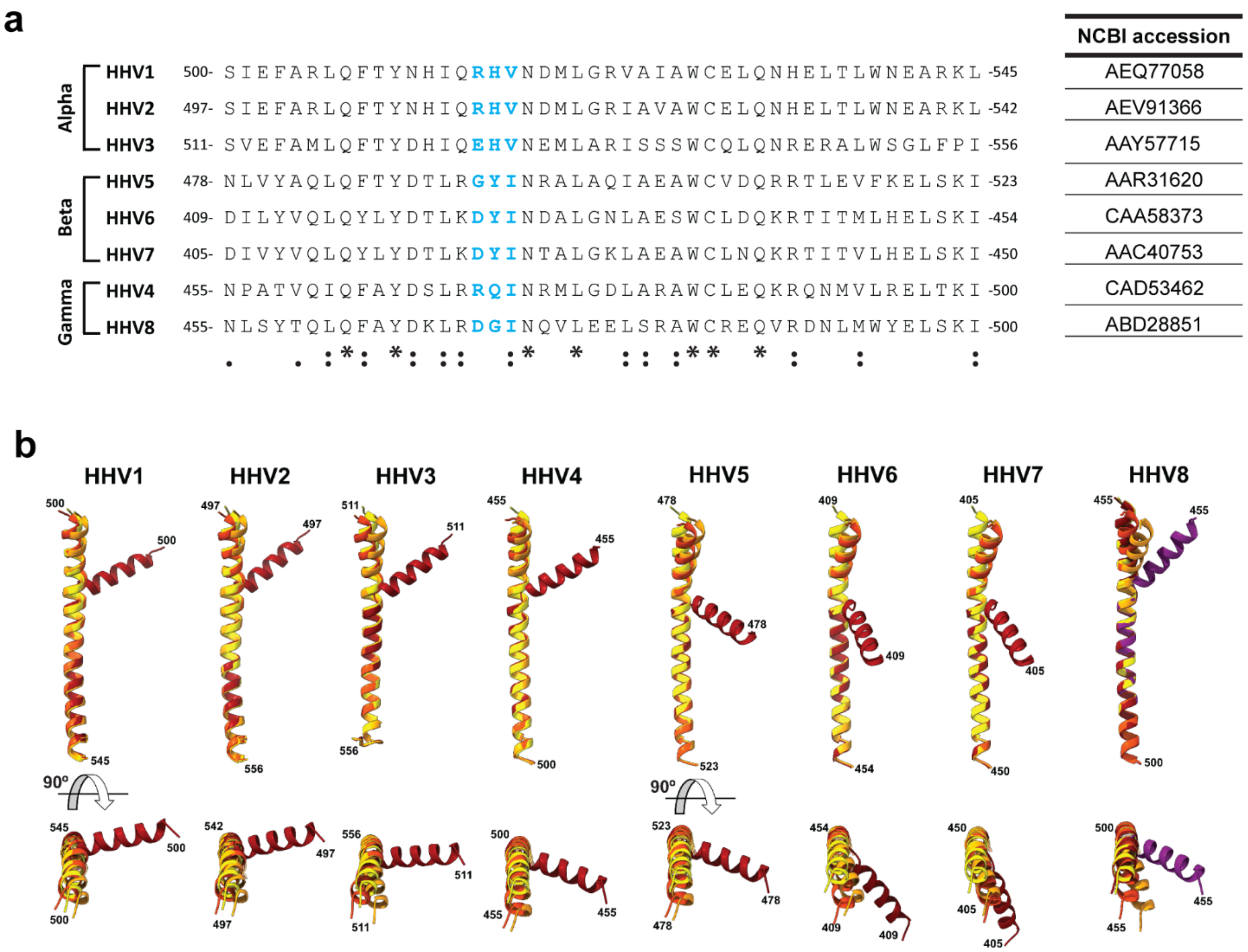

Supplementary Fig. 6. Predicted structural deformations induced by proline substitutions to residues corresponding to VZV ^{526}EHV^{528} residues in gB DIII central helix of the eight human herpesviruses. a Sequence alignment of the amino acids equivalent to VZV gB DIII central helix from human herpesviruses. gB NCBI accession are listed. Residues corresponding to VZV ^{526}EHV^{528} are colored in blue. Conservation among residues is presented based on Clustal Omega: "." indicates weak similarity; ":" indicates strong similarity; "*" indicates full conservation. **b** All structures for the wild type (WT) and mutant the gB DIII central helices were predicted using AlphaFold2 [3,4] and the mutant structures aligned to WT gB using Matchmaker [5]. WT gB is colored yellow and each of the proline substitutions is colored as for VZV gB E526 (orange), H527 (orange red), V528 (maroon). For HHV8, a fourth proline mutant, gB[G471Y/I472P] (purple) was modeled to assess the effect of tyrosine at the -1 position for I472, which had an RMSD of 7.109. The amino acids at the beginning and end of the gB DIII central helix are indicated. HHV1: HSV-1; HHV2: HSV-2; HHV3: VZV; HHV4: EBV; HHV5: HCMV; HHV8: KSHV.

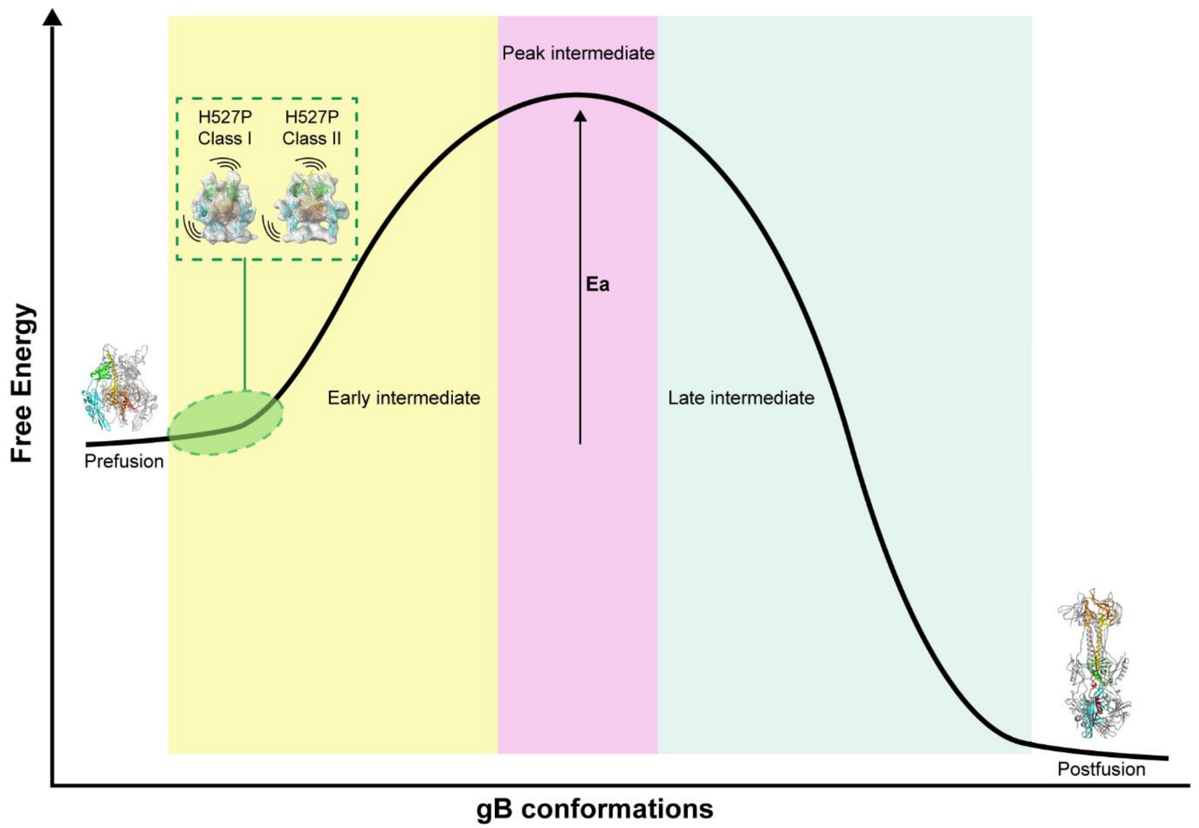

Supplementary Fig. 7. The proposed energy landscape of gB conformations for membrane fusion. The height of the free energy barrier corresponds to the effective activation energy (Ea) of the overall fusion process. The two endpoint states (prefusion and postfusion) of gB are depicted at the extremes of the curve; VZV prefusion gB based on homology modelling of HSV-1 prefusion gB[H516P] [6], and the 2.8Å VZV postfusion gB [1]. Theoretical fusion intermediates are indicated by three phases, early (yellow), peak (pink), and late (blue). The two classes of VZV gB[H527P] reported in the current manuscript are proposed to be arrested at either a prefusion-like state or very early fusion intermediate states (highlighted by the green oval). The relative energies for each entity are drawn for illustration purposes only.

Supplementary Table 1. Cryo-ET data collection parameters for WT- and H527P-gB EVs.

Parameter	Structure		
	**WT gB[A]**	**gB[H527P][A]**	**gB[H527P][B]**
**Data collection and processing**			
Microscope	Titan Krios (FEI; D3690)	Titan Krios (FEI; D3690)	Titan Krios (FEI; D3690)
Voltage (kV)	300	300	300
Magnification (1000X)	81	81	81
Spot size	6	6	5
Energy filter slit width (eV)	20	20	20
Detector	Gatan K3	Gatan K3	Gatan K3
Defocus range (μm)	-2.0	-2.0	-2.0
Pixel size (Å)	1.095	1.095	1.095
Tilt axis (°)	85.6	85.6	85.6
Tilt range (°)	± 60 (bi-directional)	± 60 (bi-directional)	± 60 (bi-directional)
Tilt increment	3°	3°	3°
Exposure time (s)	0.2 / 0.25	0.2	0.3
Frames	4	4	6
Electron exposure rate ($e^-/Å^2/s$)	11.9	11.9	10.73
Dose per frame ($e^-/Å^2$/frame)	0.573	0.573	0.54
Dose per tilt ($e^-/Å^2$)	3.44 / 4.3	3.44	3.22
Total electron exposure ($e^-/Å^2$)	141 / 176.3	93.973	132
Tomograms collected (no.)	10	17	43
**Tomogram Reconstruction**			
Symmetry imposed			3
Final particle images (no.)	N/A	N/A	2,074
Map resolution (Å)	N/A	N/A	9.8-12.4Å
FSC threshold	N/A	N/A	0.143

[A] EVs produced using WT gB and gB[H527P] pCAGGs expression constructs.
[B] EVs produced using gB[H527P]-His pCAGGs expression constructs.

Supplementary Table 2. Comparison of the structure deviation for proline and alanine mutants in the VZV gB DIII central helix predicted with AlphaFold2.

Mutant	RMSD (Pruned)[A]		RMSD (All)[A]	
	Å	AA	Å	AA
WT-DIII[B]	0.693	35	2.672	46
E526A[B]	0.926	43	1.932	46
E526P[B]	1.019	35	2.691	46
H527A[B]	0.848	43	1.909	46
H527P[B]	0.964	26	3.428	46
V528A[B]	0.937	43	1.944	46
V528P[B]	0.812	24	9.115	46
E526A[C]	0.553	46	0.553	46
E526P[C]	0.693	39	1.481	46
H527A[C]	0.730	45	0.813	46
H527P[C]	0.636	38	1.831	46
V528A[C]	0.651	46	0.651	46
V528P[C]	0.334	26	10.673	46

[A] RMSD calculated for comparisons between WT-DIII and mutant gB DIII helices using MatchMaker [5] (Chimera) Needleman-Wunsch alignment algorithm (BLOSUM-62 matrix). The RMSD values are given for the pruned sequences and the complete helix (All).

[B] Wild type gB DIII (AA511-556; WT-DIII) and DIII mutant helices predicted with AlphaFold2 compared to the structure of gB solved by cryo-EM (PDB 6VN1; https://doi.org/10.2210/pdb6VN1/pdb).

[C] DIII mutant helices predicted with AlphaFold2 compared to wild type gB DIII predicted with AlphaFold2.

Supplementary Table 3. Comparison of the structure deviation for proline mutants in the gB DIII central helix for all human herpesviruses predicted with AlphaFold2.

Virus	Alias	Strain[A]	Residue[B] [RMSD[C]]		
HHV1	HSV-1	17	R515 [0.760]	H516 [0.617]	V528 [11.016]
HHV2	HSV-2	HG52	R512 [0.857]	H513 [0.749]	V514 [9.117]
HHV3	VZV	Dumas	E526 [1.535]	H527 [1.090]	V528 [8.612]
HHV4	EBV	B95A	R470 [2.413]	Q471 [2.278]	I472 [9.099]
HHV5	HCMV	Merlin	G493 [2.355]	Y494 [2.870]	I495 [14.969]
HHV6A	-	Uganda	D424 [2.254]	Y425 [2.915]	I426 [16.107]
HHV6B	-	Z29	D424 [2.674]	Y425 [2.815]	I426 [16.416]
HHV7	-	RK	D420 [2.656]	Y421 [3.297]	I422 [16.160]
HHV8	KSHV	GK18	D470 [4.178]	G471 [2.420]	I472 [0.395][D]

[A] The strain used was the type species for each human herpesvirus.

[B] The residue in the gB DIII central helix substituted with proline.

[C] RMSD calculated for comparisons between the WT-DIII for each human herpesvirus and mutant gB DIII helices using MatchMaker (Chimera) Needleman-Wunsch alignment algorithm (BLOSUM-62 matrix). The RMSD values are given for the 46 amino acids for the entire helix.

[D] A fourth mutant was generated, G471Y/I472P, to demonstrate the effect of the glycine residue on the lack of gross structural change for the KSHV gB DIII I472P mutant. The KSHV gB[G471Y/I472P] mutant was predicted to have a RMSD of 7.109.

Supplementary Table 4. Yield efficiency of the FC-TEM experiment.

BAC	BAC transfected cells	GFP+ cells recovered by FC sorting	Cells screened by TEM	Virus replicating cells identified by TEM
pOka-TKGFP	8.7×10^6	87885	1500	1
pOka-TKGFP-gB[E526P]	8.7×10^6	40000	500	1
pOka-TKGFP-gB[H527P]	8.7×10^6	67675	2096	2
pOka-TKGFP-gB[V528P]	8.7×10^6	46662	1026	1

References

1 Oliver, S. L. *et al.* A glycoprotein B-neutralizing antibody structure at 2.8 A uncovers a critical domain for herpesvirus fusion initiation. *Nat Commun* **11**, 4141, doi:10.1038/s41467-020-17911-0 (2020).

2 Oliver, S. L. *et al.* The N-terminus of varicella-zoster virus glycoprotein B has a functional role in fusion. *PLoS Pathog* **17**, e1008961, doi:10.1371/journal.ppat.1008961 (2021).

3 Varadi, M. *et al.* AlphaFold Protein Structure Database: massively expanding the structural coverage of protein-sequence space with high-accuracy models. *Nucleic Acids Res* **50**, D439-D444, doi:10.1093/nar/gkab1061 (2022).

4 Jumper, J. *et al.* Highly accurate protein structure prediction with AlphaFold. *Nature* **596**, 583-589, doi:10.1038/s41586-021-03819-2 (2021).

5 Pettersen, E. F. *et al.* UCSF ChimeraX: Structure visualization for researchers, educators, and developers. *Protein Sci* **30**, 70-82, doi:10.1002/pro.3943 (2021).

6 Vollmer, B. *et al.* The prefusion structure of herpes simplex virus glycoprotein B. *Sci Adv* **6**, doi:10.1126/sciadv.abc1726 (2020).
