# Supplementary material for: Targeted mutagenesis of the herpesvirus fusogen central helix captures transition states
Source: Nat Commun. 2023 Dec 2;14:7958. doi: 10.1038/s41467-023-43011-w (PMC10693595; doi:10.1038/s41467-023-43011-w)
Supplement: Supplementary file 4 — Reporting Summary [file 41467_2023_43011_MOESM4_ESM.pdf]

nature portfolio

Corresponding author(s): Momei Zhou

Last updated by author(s): Oct 10, 2023

Reporting Summary

Nature Portfolio wishes to improve the reproducibility of the work that we publish. This form provides structure for consistency and transparency in reporting. For further information on Nature Portfolio policies, see our Editorial Policies and the Editorial Policy Checklist.

Statistics

For all statistical analyses, confirm that the following items are present in the figure legend, table legend, main text, or Methods section.

n/a	Confirmed	
☐	☒	The exact sample size (*n*) for each experimental group/condition, given as a discrete number and unit of measurement
☐	☒	A statement on whether measurements were taken from distinct samples or whether the same sample was measured repeatedly
☐	☒	The statistical test(s) used AND whether they are one- or two-sided<br>*Only common tests should be described solely by name; describe more complex techniques in the Methods section.*
☒	☐	A description of all covariates tested
☐	☒	A description of any assumptions or corrections, such as tests of normality and adjustment for multiple comparisons
☐	☒	A full description of the statistical parameters including central tendency (e.g. means) or other basic estimates (e.g. regression coefficient) AND variation (e.g. standard deviation) or associated estimates of uncertainty (e.g. confidence intervals)
☐	☒	For null hypothesis testing, the test statistic (e.g. *F*, *t*, *r*) with confidence intervals, effect sizes, degrees of freedom and *P* value noted<br>*Give P values as exact values whenever suitable.*
☒	☐	For Bayesian analysis, information on the choice of priors and Markov chain Monte Carlo settings
☒	☐	For hierarchical and complex designs, identification of the appropriate level for tests and full reporting of outcomes
☒	☐	Estimates of effect sizes (e.g. Cohen's *d*, Pearson's *r*), indicating how they were calculated

Our web collection on statistics for biologists contains articles on many of the points above.

Software and code

Policy information about availability of computer code

Data collection	Leica LAS X Premium, AxioVision program SE64, JEM-1400Flash, Biotek Gen5, SerialEM, FlowJo Collector's Edition.
Data analysis	WARP, EMAN2.99, Visual Molecular Dynamic (VMD 1.9.4fa29), Nanoscale Molecular Dynamics (NAMD 2.14), UCSF ChimeraX 1.14, AlphaFold2, Graphpad Prism 9.5.1, FlowJo_v10.8.1, ImageJ, Adobe Photoshop CS6.

For manuscripts utilizing custom algorithms or software that are central to the research but not yet described in published literature, software must be made available to editors and reviewers. We strongly encourage code deposition in a community repository (e.g. GitHub). See the Nature Portfolio guidelines for submitting code & software for further information.

Data

Policy information about availability of data

All manuscripts must include a data availability statement. This statement should provide the following information, where applicable:

- Accession codes, unique identifiers, or web links for publicly available datasets
- A description of any restrictions on data availability
- For clinical datasets or third party data, please ensure that the statement adheres to our policy

The data supporting the findings of this study are available within the paper and its supplementary information. Source data are provided with this paper. Each data point corresponding to figures and western blots that describe the results are provided as separate source data for Figs. 3c, 3d, 3e, 5b, S2b, S2d, S2e, S4c. All primary data will be provided by the corresponding author upon reasonable request.

Research involving human participants, their data, or biological material

Policy information about studies with human participants or human data. See also policy information about sex, gender (identity/presentation), and sexual orientation and race, ethnicity and racism.

Reporting on sex and gender	N/A
Reporting on race, ethnicity, or other socially relevant groupings	N/A
Population characteristics	N/A
Recruitment	N/A
Ethics oversight	N/A

Note that full information on the approval of the study protocol must also be provided in the manuscript.

Field-specific reporting

Please select the one below that is the best fit for your research. If you are not sure, read the appropriate sections before making your selection.

☒ Life sciences ☐ Behavioural & social sciences ☐ Ecological, evolutionary & environmental sciences

For a reference copy of the document with all sections, see nature.com/documents/nr-reporting-summary-flat.pdf

Life sciences study design

All studies must disclose on these points even when the disclosure is negative.

Sample size	No statistical method was used to predetermine sample size. For transfection-based studies, a minimum of two clones were evaluated in quantitative experiments. For VZV infection-based studies, a minimum of two experiments were performed with three biological replicates. Sample sizes were sufficient as statistical significance was reached using one-way ANOVA.
Data exclusions	Data were not excluded from the analyses.
Replication	All experiments were performed at minimum in two independent experiments, with at least two biological replicates. All attempts at replication for all the experiments were reproducible.
Randomization	Randomization was not performed as quantitative data were derived from cell culture-based experiments, but independent cell cultures or passages were used for independent replication on different days.
Blinding	The corresponding author planned and performed all quantitative experiments, so blinding of the data was not performed.

Reporting for specific materials, systems and methods

We require information from authors about some types of materials, experimental systems and methods used in many studies. Here, indicate whether each material, system or method listed is relevant to your study. If you are not sure if a list item applies to your research, read the appropriate section before selecting a response.

Materials & experimental systems

n/a	Involved in the study	
☐	☒	Antibodies
☐	☒	Eukaryotic cell lines
☒	☐	Palaeontology and archaeology
☒	☐	Animals and other organisms
☒	☐	Clinical data
☒	☐	Dual use research of concern
☒	☐	Plants

Methods

n/a	Involved in the study	
☒	☐	ChIP-seq
☐	☒	Flow cytometry
☒	☐	MRI-based neuroimaging

Antibodies

Antibodies used	Human mAb anti-gB 93k (purified in house 1mg/ml) was used in immunofluorescence (1:200), and was conjugated with Cy5

Antibodies used	(conjugation was done in house using Lightning-Link@Rapid Cy5 antibody labeling kit) and was used in flow cytometry (1:100); Mouse mAb anti-gB SG2-2E6 (1.15 mg/ml, Meridian Life Sciences) was used in immunofluorescence (1:200), and was cross-linked to protein A beads (20ug/30ul beads) in house, used in immunoprecipitation (30ul beads slurry per 400ul cell lysate); Rabbit polyclonal anti-gB antibody 746-868 (Oliver et al., 2009. J. Virol) was used in western blot (1:4,000); mouse mAb anti-GAPDH (Sigma-Aldrich Chemie GmbH, CL3266, 1 µg/ml) was used in western blot; rabbit anti-His6 antibody (ab9108, Abcam, 1 µg/ml) was used in western blot. VZV mouse mixed mAb (Meridian Life Sciences C05108MA) was used for immunohistochemistry (1:2,000), followed by biotinylated anti-mouse IgG (Vector Laboratories #BA-9200, 1: 1,000), and alkaline phosphatase streptavidin (Jackson ImmunoResearch #016-050-084, 1: 400); mouse mAb anti-VZV IE62 (EMD Millipore MAB8616) was used in immunofluorescence (1:200); rabbit polyclonal anti-VZV capsid-ORF23 (Chaudhuri et al., 2008. J. Virol) was used in immunofluorescence (1:200); rabbit polyclonal anti-EEA1 (NBP1-30914, Novus Biological) was used in immunofluorescence (1:200); sheep polyclonal anti-TGN46 (AHP500G,BioRad) was used in immunofluorescence (1:250).
Validation	All antibodies were validated for each experimental setting for optimum dilution prior to use for the acquisition of quantitative data. The specificity of antibodies were determined by a lack of cross-reactivity with non-specific antigen. The specificity of human mAb 93k and rabbit polyclonal antibody 746-868 have been demonstrated in previous publications (Oliver et al., 2020. Nature Communications and Oliver et al., 2009. J. Virol). The specificity of mouse mAb anti-gB SG2-2E6 has been demonstrated in previous publications (Yang et al., 2016. J. Virol; Oliver et al., 2020. Nature communications; Oliver et al., 2021. Plos Pathogens). The specificity of mouse mAb anti-GAPDH has been stated as "enhanced validation by RNAi knockdown" on Sigma-Aldrich website. The specificity of rabbit anti-His6 antibody has been verified in "ELISA: Antibody specificity was verified by ELISA against peptide conjugated to BSA (HHHHHH-BSA). A 1:25,000 dilution of the antibody gave an OD=1.0 in a 15 minute reaction using HRP-conjugated Goat anti-Rabbit IgG at 1:20,000 and TMB as the substrate. Appropriate specificity controls were run" on Abcam website. VZV mouse mixed mAb (Meridian Life Sciences C05108MA) specificity has been demonstrated in previous publications (Sen et al., 2011. PNAS; Zhou et al., 2020. Plos Pathogens). The specificity of mouse mAb anti-VZV IE62 was stated as "Recognizes the VZV immediate early gene 62. No cross-reactivity to HSV" on Millipore website. Rabbit polyclonal anti-VZV capsid-ORF23 specificity has been demonstrated in previous publications (Chaudhuri et al., 2008. J. Virol; Yang et al., 2014. Plos Pathogens). The specificity of rabbit polyclonal anti-EEA1 has been demonstrated in previous publications (Yang et al., 2014. Plos Pathogens; Olive et al., 2013. PNAS). The specificity of sheep polyclonal anti-TGN46 has been demonstrated in previous publications (Hauser et al., 2010. Retrovirology; Yang et al., 2014. Plos Pathogens).

Eukaryotic cell lines

Policy information about cell lines and Sex and Gender in Research

Cell line source(s)	MeWo-DSP2 cells were derived from human melanoma MeWo cells originally from ATCC (HTB-65); CHO-DSP1 cells were derived from Chinese Hamster Ovary (CHO) K1 cells originally from ATCC (CCL-61). MeWo-DSP2 and CHO-DSP1 were developed in house (Yang et al., 2016, J. Virol). Baby hamster kidney (BHK-21) cells were originally from ATCC (C-13;CCL-10).
Authentication	MeWo cells and BHK-21 cells were not authenticated further. The MeWo-DSP2 and CHO-DSP1 cells were authenticated in experimental assays using positive controls that demonstrated the reconstitution of the split luciferase-green fluorescent protein (GFP) DSP1 and DSP2 to yield luciferase activity and GFP signal. The last authentication was performed as the last stable reporter fusion assay (SRFA) on January-05-2022, and the results showed that both cell lines constitutively expressed DSP proteins.
Mycoplasma contamination	Cell lines have not recently been tested for mycoplasma contamination.
Commonly misidentified lines (See ICLAC register)	Commonly misidentified lines were not used.

Flow Cytometry

Plots

Confirm that:

☒ The axis labels state the marker and fluorochrome used (e.g. CD4-FITC).

☒ The axis scales are clearly visible. Include numbers along axes only for bottom left plot of group (a 'group' is an analysis of identical markers).

☒ All plots are contour plots with outliers or pseudocolor plots.

☒ A numerical value for number of cells or percentage (with statistics) is provided.

Methodology

Sample preparation	CHO-DSP1 cells were transfected with VZV glycoprotein gB Wild type or mutants, or gH/gL non-specific antigen control.
Instrument	DXP multi-color FACScan analyzer (Cytek Biosciences).
Software	FlowJo Collector's Edition was used as the software for data acquisition, and FlowJo_v10.8.1 was used for data analysis.
Cell population abundance	Only one cell type CHO-DSP1 was present, and 20,000 events were collected and recorded.

Gating strategy

FSC and SSC were used to gate on non-debris single cell population. gB-expressing positive values were determined from cells transfected with non-specific antigen control, gH/gL.

☒ Tick this box to confirm that a figure exemplifying the gating strategy is provided in the Supplementary Information.
